# Complete Genome Sequence of a Novel Multidrug-Resistant *Klebsiella pneumoniae* Phage, vB_Kpn_IME260

**DOI:** 10.1128/genomeA.00055-17

**Published:** 2017-05-11

**Authors:** Shaozhen Xing, Xiangchun Pan, Qiang Sun, Guangqian Pei, Xiaoping An, Zhiqiang Mi, Yong Huang, Baohua Zhao, Yigang Tong

**Affiliations:** a Hebei Normal University, College of Life Science, Hebei, China; b State Key Laboratory of Pathogen and Biosecurity, Beijing Institute of Microbiology and Epidemiology, Beijing, China

## Abstract

*Klebsiella pneumoniae* is the most common clinically important opportunistic bacterial pathogen and its infection is often iatrogenic. Its drug resistance poses a grave threat to public health. The genomic data reported here comprise an important resource for research on phage therapy in the control of drug-resistant bacteria.

## GENOME ANNOUNCEMENT

*Klebsiella pneumoniae* is an important opportunistic pathogenic bacterium, and its infection is often iatrogenic; it readily infects the respiratory tract, urogenital tract, and bloodstream (1). In recent years, the abuse of antibiotics has led to a dramatic increase in the resistance of *K. pneumoniae*, making clinical treatment increasingly difficult (2). Phages have unparalleled advantages in the treatment of bacterial infections, especially antibiotic-resistant infections (3). A novel T5-like *K. pneumoniae* phage, vB_Kpn_IME260, was isolated from sewage water collected from the 307th Hospital of the Chinese People’s Liberation Army, Beijing, China. This phage was found to efficiently lyse antibiotic-resistant *K. pneumoniae* strains 465, 495, 501, 1731, 1732, 1733, 1734, 1735, and 1736, which had also been isolated from this hospital.

The bacteriophage genomic DNA was extracted using the proteinase K/SDS method (4). Next-generation sequencing (NGS) was used to sequence the complete genome of bacteriophage vB_Kpn_IME260 with a MiSeq PE300 sequencer (Illumina, USA). The average read length of the raw sequencing reads was 298.79 bases, with a total number of 94,072. Roche Newbler version 3.0 software was used to assemble the NGS reads of vB_Kpn_IME260, which resulted in a circular phage genome contig with a length of 113,213 bp and approximately 224× coverage. Its genome size was found to be 123,490 bp, with a directed terminal repeat of 10,277 bp. The genomic termini were predicted based on NGS data (5), and the lengths of the predicted termini were similar to the terminal lengths of other T5-like phages. The coverage of the terminal sequence region was significantly higher than other genome regions, which also suggested that the prediction of the termini was reliable. The genome of vB_Kpn_IME260 has a G+C content of 45.5%. The BLASTn results showed that vB_Kpn_IME260 was most similar to the *Salmonella* phage Stitch (GenBank: KJ190157.1) with 14% genome coverage and 74% sequence identity. Results from the Rapid Annotations using Subsystems Technology server predicted 171 open reading frames (ORFs), which included 61 functional ORFs with 22 tRNAs and 88 ORFs encoding hypothetical proteins (6). These genomic data comprise an important resource for research on the functions of hypothetical proteins and phage lysozymes in controlling specific bacterial species.

### Accession number(s).

The whole-genome sequence of *K. pneumoniae* phage vB_Kpn_IME260 has been deposited in GenBank under the accession number KX845404.
